# Microarray based global transcriptome profiling reveals involvement of non-Hsa21 genes and microRNAs in molecular mechanism of Down syndrome pathogenesis

**DOI:** 10.1186/1755-8166-7-S1-P132

**Published:** 2014-01-21

**Authors:** Ashutosh Pathak, Divya Agarwal, Shubha R Phadke

**Affiliations:** 1 Department of Medical Genetics, Sanjay Gandhi Post Graduate Institute of Medical Sciences, Lucknow, India

## 

Down syndrome (DS), the most frequent genetic disorder leading to mental retardation is caused by partial/complete triplication of human chromosome 21 (Hsa21). The differential expression of genes located on extra chromosome 21 is assumed responsible for phenotypic abnormalities but this gene dosage hypothesis has not been assessed on genome-wide basis. The gene expression patterns related to phenotypic abnormalities may provide insights into their roles in DS pathogenesis. To analyze the differential gene expression and understand the molecular mechanism underlying pathogenesis of DS, we performed global gene expression profiling in blood samples of 14 DS and 4 normal subjects using human whole transcriptome microarray. The microarray analysis revealed total of 624 genes (195 upregulated and 429 down regulated) were differentially expressed in DS patients as compared to control. Out of the genes present on chromosome-21, a total of 210 genes were differentially expressed ranging from 1.5 to 5 fold compared to normal individuals. Genes involved in physiological pathways such as apoptosis and cell cycle regulation, signal transduction, cell maturation, and immunity showed dysregulation. Several genes localized on Hsa21 such as *APP*, *SOD*1, *DYRK*1A, *COL*6A1 showed differential expression and the levels were conserved across all DS subjects. Interestingly, several non-Hsa21 genes such as *RCAN*3 (Hsa1), *ANK*3 (Hsa10), *CDK*17 (Hsa12) etc., having roles in cardiogenesis, signal transduction and differentiation of neurons showed conserved levels of expression across the DS subjects. Further to investigate the role of microRNAs (miRNAs) in regulation of gene expression, global miRNA profiling was performed in 4 DS patients and 1 control using Affymetrix miRNA 3.0 array. Several Hsa21 miRNAs like miR-99a, let-7c, miR-125b-2, miR155, miR-802 showed overexpression effecting the regulation of genes involved in DS pathogenesis. Our results substantiate that involvement of non-Hsa21 genes and miRNAs provide etiological basis for abnormal phenotypes during DS pathogenesis. Our data lead to more systematic understanding of molecular mechanism underlying DS pathogenesis and identification of miRNA based therapeutic targets for better management of the disease.

